# Evidence that complement and coagulation proteins are mediating the clinical response to omega-3 fatty acids: A mass spectrometry-based investigation in subjects at clinical high-risk for psychosis

**DOI:** 10.1038/s41398-022-02217-0

**Published:** 2022-10-28

**Authors:** Subash Raj Susai, Colm Healy, David Mongan, Meike Heurich, Jonah F. Byrne, Mary Cannon, Gerard Cagney, Kieran Wynne, Connie Markulev, Miriam R. Schäfer, Maximus Berger, Nilufar Mossaheb, Monika Schlögelhofer, Stefan Smesny, Ian B. Hickie, Gregor E. Berger, Eric Y. H. Chen, Lieuwe de Haan, Dorien H. Nieman, Merete Nordentoft, Anita Riecher-Rössler, Swapna Verma, Rebekah Street, Andrew Thompson, Alison Ruth Yung, Barnaby Nelson, Patrick D. McGorry, Melanie Föcking, G. Paul Amminger, David Cotter

**Affiliations:** 1 Department of Psychiatry, RCSI University of Medicine and Health Sciences, Dublin, Ireland; 2 School of Pharmacy and Pharmaceutical Sciences, Cardiff University, Cardiff, UK; 3 School of Biomolecular and Biomedical Science, Conway Institute, University College Dublin, Dublin, Ireland; 4 Systems Biology Ireland, University College Dublin, Dublin, Ireland; 5 Centre for Youth Mental Health, The University of Melbourne, Melbourne, VIC Australia; 6 Orygen, 35 Poplar Rd, Parkville, VIC 3052 Australia; 7 Department of Psychiatry and Psychotherapy, University of Bern, Bern, Switzerland; 8 Department of Psychiatry and Psychotherapy, Medical University of Vienna, Vienna, Austria; 9 BioPsyC-Biopsychosocial corporation – Non-Profit association for research funding, Vienna, Austria; 10 Department of Psychiatry and Psychotherapy, Jena University Hospital, Jena, Germany; 11 Brain and Mind Centre, The University of Sydney, Sydney, NSW Australia; 12 Child and Adolescent Psychiatric Service of the Canton of Zurich, Zürich, Switzerland; 13 Department of Psychiatry, University of Hong Kong, Pok Fu Lam, Hong Kong; 14 Department of Psychiatry, Academic Medical Center, Amsterdam, The Netherlands; 15 Mental Health Center Copenhagen, Department of clinical Medicine, Copenhagen University Hospital, Copenhagen, Denmark; 16 Medical Faculty, University of Basel, Basel, Switzerland; 17 Institute of Mental Health, Singapore, Singapore; 18 Institute for Mental and Physical Health and Clinical Translation (IMPACT), Deakin University, Geelong, VIC Australia; 19 School of Health Sciences, University of Manchester, Manchester, UK

**Keywords:** Schizophrenia, Molecular neuroscience

## Abstract

Preliminary evidence indicates beneficial effects of omega-3 polyunsaturated fatty acids (PUFAs) in early psychosis. The present study investigates the molecular mechanism of omega-3 PUFA-associated therapeutic effects in clinical high-risk (CHR) participants. Plasma samples of 126 CHR psychosis participants at baseline and 6-months follow-up were included. Plasma protein levels were quantified using mass spectrometry and erythrocyte omega-3 PUFA levels were quantified using gas chromatography. We examined the relationship between change in polyunsaturated PUFAs (between baseline and 6-month follow-up) and follow-up plasma proteins. Using mediation analysis, we investigated whether plasma proteins mediated the relationship between change in omega-3 PUFAs and clinical outcomes. A 6-months change in omega-3 PUFAs was associated with 24 plasma proteins at follow-up. Pathway analysis revealed the complement and coagulation pathway as the main biological pathway to be associated with change in omega-3 PUFAs. Moreover, complement and coagulation pathway proteins significantly mediated the relationship between change in omega-3 PUFAs and clinical outcome at follow-up. The inflammatory protein complement C5 and protein S100A9 negatively mediated the relationship between change in omega-3 PUFAs and positive symptom severity, while C5 positively mediated the relationship between change in omega-3 and functional outcome. The relationship between change in omega-3 PUFAs and cognition was positively mediated through coagulation factor V and complement protein C1QB. Our findings provide evidence for a longitudinal association of omega-3 PUFAs with complement and coagulation protein changes in the blood. Further, the results suggest that an increase in omega-3 PUFAs decreases symptom severity and improves cognition in the CHR state through modulating effects of complement and coagulation proteins.

## Introduction

The brain is a lipid-rich organ and 60% of its total membrane is composed of phospholipids [1]. Polyunsaturated Fatty acids (PUFAs) are a vital component of neuronal membrane phospholipids. Omega-3 and omega-6 fatty acids are two major classes of PUFAs present in the brain, among which omega-3 PUFAs have superior health benefits in humans [2–6]. Pre-clinical investigations have identified several biological mechanisms in which omega-3 PUFAs play an important role, such as the maintenance of cell membrane integrity [7, 8], release of specialized pro-resolving mediators [9–12], modification of gut microbiome [13] and regulation of synaptic pruning activity in the brain [14–16].

In psychotic disorder, existing literature has indicated an association of PUFA-related dietary factors such as insufficient consumption of omega-3 PUFAs [17–19] or abnormal fat metabolism [20–27] with the occurrence of psychotic symptoms. The membrane phospholipid hypothesis of schizophrenia proposes a possible link between PUFA abnormalities and psychosis and suggests a potential therapeutic role of omega-3 PUFAs in the treatment of schizophrenia and related disorders at an early stage [20, 28–40]. To date, the evidence for the therapeutic role of omega-3 PUFAs in the Clinical High Risk (CHR) population appears inconclusive. The first omega-3 fatty acid placebo-controlled randomized UHR trial the Vienna High Risk study found a large preventive effect on transition rate [41] while a consecutive multi-center replication study (the NEURAPRO trial) was not able to confirm these prior finding [42, 43]. The Vienna High Risk trial found a reduction in phospholipase A2 (PLA2) activity in relation to omega-3 PUFAs at 12 weeks follow-up [44]. PLA2 is involved in fatty acid metabolism which is found to be increased in schizophrenia patients [45]. Furthermore, omega-3 PUFA supplementation also indicated an increase in soluble intercellular adhesion molecule-1 (sICAM-1) in Vienna High Risk study participants [46]. In contrast, in the NEURAPRO study, an inverse relationship between omega-3 PUFAs and plasma cytokines was found, but this association did not indicate any clinical benefits on psychopathology of CHR participants [47]. Knowledge of the underlying mechanism of omega-3 PUFAs will provide an insight into the biological role of omega-3 PUFAs in the pathophysiology of psychosis and help in designing early intervention strategies [41, 48, 49].

The current study investigates the relationship of PUFAs (omega-3 and omega-6) with plasma proteomic pathways in a clinical CHR population. We performed mass spectrometry-based analysis on plasma samples at baseline and follow-up, to investigate the longitudinal association between PUFAs and the plasma proteome. Furthermore, we evaluated the proteomic pathways through which omega-3 PUFAs may influence psychopathology in CHR participants. Thus, through this study we addressed the following research questions:

I. Are there any associations between changes in omega-3 PUFAs with plasma proteins at follow-up in CHR participants?
II. Which biological pathways are most substantially influenced by change in PUFAs (both omega-3 and omega-6 PUFAs)?
III. Do the identified plasma proteins mediate the relationship between change in omega-3 PUFAs and clinical outcomes?

## Materials and methods

### Study participants

The NEURAPRO study is a multicentre randomized placebo-controlled clinical trial registered with the Australian New Zealand Clinical Trial Registry as ACTRN 12608000475347. The study was performed abiding with the Declaration of Helsinki [50] and adhering to the National Health and Medical Research Council of the Australia National Statement on Human Research. The trial aimed to evaluate the therapeutic role of omega-3 PUFAs in preventing the development of psychosis in CHR patients. Informed consent was obtained from all the participants or from their parents/guardians if they were younger than 17 years. The inclusion and exclusion criteria of the participants of the study are provided in [51].

The participants received either omega-3 PUFAs (840 mg eicosapentaenoic acid [EPA] and 560 mg docosahexaenoic acid [DHA] per day) or placebo (an equivalent dose of paraffin oil) for 6 months [43]. The adherence to the study interventions was assessed monthly. At the end of 6 months, a low adherence of 43% to the omega-3 intervention and a 41% to the placebo was reported [52].

### Measurement of omega-3 PUFAs

Fasting blood samples were collected at baseline and 6-month follow-up. The molecular percentage of the total sum of the omega-3 and omega-6 fatty acids in erythrocyte membrane rafts were measured based on the phosphatidyl-ethanolamine fraction using gas chromatography [53]. Total omega-3 PUFAs comprise of alpha linolenic acid (18:3), eicosapentaenoic acid (20:5), docosapentaenoic acid (22:5), and docosahexaenoic acid (22:6). Total omega-6 PUFAs include linoleic acid (18:2), gamma-linoleic acid (18:3), eicosadienoic acid (20:2), dihomo gamma-linoleic acid (20:3), arachidonic acid (20:4) and adrenic acid (22:4). Since a poor adherence to the study intervention was observed in both study arms, the erythrocyte membrane levels were used as objective measure of dietary intake of PUFAs (exposure variable) [54, 55].

### Quantification of plasma proteome

Plasma samples of baseline and follow-up time points were used for discovery-based, data-dependant acquisition (DDA), mass spectrometry. For sample preparation steps and mass spectrometry protocols refer to Supplementary Methods.

### Clinical outcome measures

The clinical outcomes of psychotic symptom severity (PSS), functional status, and cognitive status at 6-months follow-up were considered for the analyses. The PSS was assessed using the Comprehensive Assessment of At-Risk Mental State (CAARMS) scale [56]. The subscales of positive symptoms from the CAARMS assessment (unusual thought content, non-bizarre ideas, perceptual abnormalities, and disorganized speech) were used for the calculation of the PSS score. The summed scores of the product of global rating scale score (0–6) and frequency (0–6) of these subscales were calculated, as per previous research [57, 58]. Functional outcome was measured using the Social and Occupational Functional assessment Scale (SOFAS) and cognitive outcome using the Brief Assessment of Cognition in Schizophrenia (BACS), both at 6-months follow-up [59, 60].

### Statistical analysis

Statistical analysis was performed using IBM^®^ SPSS^®^ statistics version 26 and STATA IC^®^ version 16.

#### Analysis 1- Identification of proteins and pathways associated with change in PUFAs

Linear regression models were used to assess longitudinal associations between 6-month change in erythrocyte PUFAs (total omega-3 or total omega-6 PUFAs) and plasma proteins at follow-up. Models were adjusted for age and sex. Proteins that were significantly associated (*p* < 0.05) with change in total omega-3 and omega-6 PUFAs were then taken forward for pathway analysis. Pathway analysis was conducted using the Reactome Pathway Knowledgebase Enrichment Analysis and a probability factor (*p*-value) was generated for each pathway based on the protein representations [61]. A list of biological pathways based on the p-values after Benjamini-Hochberg correction for multiple tests (FDR 5%) was generated. The UNIPROT entities that were associated with total omega-3 PUFAs were considered for further analysis.

#### Analysis 2- Relationship of total omega-3 associated proteins and clinical outcome

The relationship of total omega-3 PUFAs associated proteins (from analysis 1) with clinical outcomes at 6-month follow-up were assessed using a linear regression model. The PSS, SOFAS, and BACS scores were used for the analysis. The models were adjusted for age, sex, and corresponding baseline protein levels.

#### Analysis 3: Univariate mediation model

**Fig. 1:**
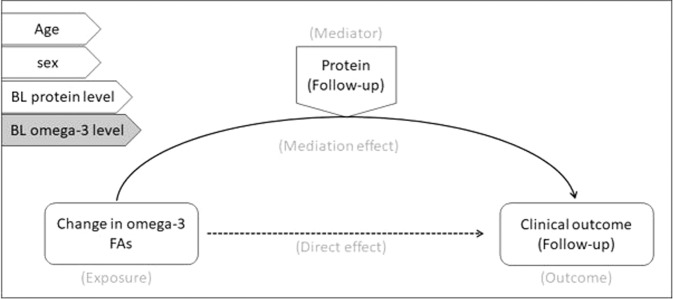
Mediation model. The general structure of the mediation model mentioning the exposure (change in omega-3 levels), mediator (protein levels) and outcome variables (clinical outcome). The model was adjusted for the covariates (age, sex, baseline protein and omega-3 levels) mentioned on the left side of the picture.

Mediation analysis was performed to evaluate the potential mediating role of plasma proteins in the relationship between total omega-3 PUFAs and clinical outcomes [62]. Regression-based mediation analysis was performed in IBM® SPSS® using the PROCESS platform. Regression beta coefficients were constructed using a conventional mediation analysis model with a bootstrap sample size of 5000 and a 95% confidence interval. In the mediation model, the change in total omega-3 levels were used as exposure variable, the protein measures at follow-up were used as mediators and the clinical and neurocognitive outcomes (PSS/SOFAS and BACS) at follow-up were used as the outcome measures. The mediation analysis was adjusted for age, sex, and corresponding baseline plasma protein levels (Fig. 1). The role of baseline total omega-3 PUFAs on the mediation model was then assessed by repeating the model with baseline total omega-3 PUFAs levels as an additional covariate.

## Results

From 285 CHR participants in the NEURAPRO trial, 146 participants provided plasma samples at both time-points, baseline, and 6-month follow-up. Out of these, 128 participants had erythrocyte omega-3 PUFA levels and proteomic measurements at both time-points. These 128 participants were considered for the statistical analysis and the baseline characteristics of these participants are given in Table 1. A total of 165 proteins from the discovery proteomics approach that passed quality control were eligible for analysis.

**Table 1:** Participants’ demographic, anthropometric, PUFA, and clinical characteristics at baseline and follow-up.

		Baseline	6-month follow-up
Demographic details			
Age in years, mean ± SD		18 ± 4	—
BMI in kg/m^2^, mean ± SD		24.20 ± 5.43	—
Gender, *n* (%)	Female	81 (63%)	—
	Male	47 (37%)	—
Biological and clinical measures			
Erythrocyte membrane fatty acid levels in %, mean ± SD	Total omega-3 fatty acids	11.94 ± 1.68	13.34 ± 4.40
	Total omega-6 fatty acids	35.56 ± 1.73	31.47 ± 4.12
Positive symptom severity (PSS) score, mean ± SD		25 ± 22	14 ± 15
Social and Occupational Functional Assessment Scale score, mean ± SD		55 ± 10	67 ± 15
Brief Assessment of Cognition in Schizophrenia- composite score, mean ± SD		25 ± 22	51 ± 13

*SD* Standard deviation.

### Results from analysis 1: The longitudinal association between change in PUFAs and plasma proteins

In a linear regression model, a 0 to 6-month change in total omega-3 PUFAs was associated with 24 plasma proteins at follow-up after adjusting for age, sex, and baseline total omega-3 levels. Using pathway analysis, these 24 proteins represented three major biological pathways, (i) the immune system, (ii) hemostasis (coagulation), and (iii) vesicle mediated transport. The complement system and sub-pathways were the top pathways denoted by the change in total omega-3 PUFA associated proteins (Table 2 and Supplementary Table 1). In the coagulation cascade pathway, the plasma proteins associated with change in omega-3 PUFAs (hereafter omega-3 related proteins) associated with platelet activation and clotting cascade-related mechanisms (Table 2 and Supplementary Table 1). In contrast to omega-3 fatty acids, the plasma proteins associated with a change in omega-6 PUFAs did not indicate any major biological pathways (Supplementary Tables 2 and 3).

**Table 2:** Pathways significantly associated with 6-month change in total omega-3 PUFAs.

Pathway name	#Entities found	#Reactions found	#Interactors found	Entities FDR
Regulation of complement cascade	10	31	6	<0.001
Complement cascade	10	49	6	<0.001
Initial triggering of complement	6	11	0	<0.001
Classical antibody-mediated complement activation	5	2	0	<0.001
Creation of C4 and C2 activators	5	2	0	<0.001
Platelet degranulation	5	2	0	<0.001
Innate immune system	14	114	8	<0.001
Binding and Uptake of Ligands by Scavenger Receptors	5	9	1	<0.001
Post-translational protein phosphorylation	4	1	0	<0.001
Response to elevated platelet cytosolic Ca2+	5	2	0	<0.001
Scavenging of heme from plasma	4	6	1	<0.001
FCGR activation	4	6	0	<0.001
Terminal pathway of complement	2	5	1	0.002
Regulation of Insulin-like Growth Factor (IGF) transport and uptake by Insulin-like Growth Factor Binding Proteins (IGFBPs)	4	1	0	0.009
Role of phospholipids in phagocytosis	4	6	1	0.012
Plasma lipoprotein assembly	3	6	1	0.013
Transport of gamma-carboxylated protein precursors from the endoplasmic reticulum to the Golgi apparatus	2	2	0	0.013
FCGR3A-mediated IL10 synthesis	4	10	1	0.013
Parasite infection	4	14	1	0.013
FCGR3A-mediated phagocytosis	4	14	1	0.013
Leishmania phagocytosis	4	14	1	0.013
Gamma-carboxylation of protein precursors	2	2	0	0.016
Formation of Fibrin Clot (Clotting Cascade)	3	14	0	0.016
Removal of aminoterminal propeptides from gamma-carboxylated proteins	2	2	0	0.017
Defective F9 secretion	1	1	0	0.021
Activation of C3 and C5	2	2	1	0.021
Gamma-carboxylation, transport, and amino-terminal cleavage of proteins	2	6	0	0.021
Regulation of actin dynamics for phagocytic cup formation	4	7	1	0.025
Chylomicron assembly	2	2	1	0.039
Intrinsic Pathway of Fibrin Clot Formation	2	5	0	0.039
Neutrophil degranulation	4	4	0	0.044
Leishmania parasite growth and survival	4	10	1	0.044
Anti-inflammatory response favouring Leishmania parasite infection	4	10	1	0.044
Fcgamma receptor (FCGR) dependent phagocytosis	4	19	1	0.048

The table shows the lists of pathways that were significantly represented by total omega-3 associated plasma proteins. The names of pathways are given in the order of *p*-values from low to high.

**Table 3:** Results of linear regression model II, showing a relationship between plasma proteins (which were showing an association with change in omega-3 FAs) with clinical outcomes at 6 months follow-up.

Clinical outcomes		PSS			SOFAS			BACS		
Protein names		Coef.	*P* value	95% Conf. interval	Coef.	*P* value	95% Conf. interval	Coef.	*P* value	95% Conf. interval
Alpha-1-antitrypsin	SERPINA1	0.41	0.78	−2.44 to 3.27	0.02	0.99	−2.73 to 2.77	−0.77	0.55	−3.32 to 1.77
Alpha-1B-glycoprotein	A1BG	0.79	0.57	−1.94 to 3.53	0.05	0.97	−2.59 to 2.69	−0.50	0.7	−3.08 to 2.08
Apolipoprotein C-I	APOC1	1.03	0.47	−1.80 to 3.86	−1.22	0.37	−3.94 to 1.49	−1.16	0.36	−3.68 to 1.35
Apolipoprotein C-III	APOC3	−0.98	0.5	−3.82 to 1.87	1.30	0.34	−1.40 to 4.01	2.85	0.03*	0.36 to 5.34
Apolipoprotein D	APOD	1.17	0.41	−1.62 to 3.96	2.77	0.04*	0.13 to 5.41	3.87	0.00*	1.25 to 6.50
Apolipoprotein E	APOE	−2.15	0.12	−4.87 to 0.58	1.37	0.3	−1.24 to 3.97	3.09	0.01*	0.71 to 5.48
Apolipoprotein L1	APOL1	1.23	0.38	−1.52 to 3.99	1.13	0.4	−1.51 to 3.78	1.96	0.12	−0.49 to 4.42
Caspase-14	CASP14	1.84	0.19	−0.95 to 4.62	1.08	0.43	−1.60 to 3.75	−1.29	0.31	−3.81 to 1.24
Coagulation factor V	F5	−0.05	0.97	−2.86 to 2.76	1.48	0.28	−1.20 to 4.16	3.67	0.00*	1.22 to 6.11
Complement C1q subcomponent subunit B	C1QB	−0.01	0.99	−2.83 to 2.80	0.95	0.49	−1.75 to 3.64	3.93	0.00*	1.58 to 6.28
Complement C5	C5	3.54	0.01*	0.79 to 6.30	−3.23	0.02*	−5.87 to −0.59	−0.88	0.49	−3.38 to 1.63
Complement component C7	C7	0.16	0.91	−2.66 to 2.98	0.06	0.97	−2.62 to 2.74	2.06	0.1	−0.39 to 4.51
Complement factor B	CFB	0.78	0.58	−2.01 to 3.58	−1.98	0.15	−4.66 to 0.71	−3.18	0.02*	−5.72 to −0.63
Complement factor I	CFI IF	2.49	0.07	−0.22 to 5.21	−2.36	0.08	−4.97 to 0.25	−0.30	0.81	−2.77 to 2.16
Filamin A-interacting protein 1-like protein	FILIP1L	0.18	0.9	−2.56 to 2.92	0.84	0.54	−1.84 to 3.53	1.20	0.34	−1.28 to 3.67
Galectin-3-binding protein	LGALS3BP	0.24	0.87	−2.67 to 3.16	−0.89	0.53	−3.68 to 1.91	0.27	0.85	−2.46 to 2.99
Haptoglobin	HP	1.07	0.45	−1.72 to 3.85	−1.34	0.32	−4.01 to 1.33	−0.58	0.65	−3.11 to 1.95
Immunoglobulin heavy constant gamma 2	IGHG2	−1.83	0.19	−4.56 to 0.89	0.81	0.54	−1.80 to 3.42	−0.85	0.5	−3.32 to 1.62
Immunoglobulin heavy constant gamma 4	IGHG4	−3.13	0.02*	−5.79 to −0.47	−0.22	0.87	−2.80 to 2.36	1.98	0.11	−0.46 to 4.42
Immunoglobulin heavy variable 1–18	IGHV1-18	0.2	0.89	−2.59 to 3.00	−0.05	0.97	−2.74 to 2.65	0.75	0.56	−1.79 to 3.29
Immunoglobulin heavy variable 3–7	IGHV3-7	−0.29	0.84	−3.05 to 2.48	0.27	0.84	−2.38 to 2.91	1.57	0.21	−0.87 to 4.01
Immunoglobulin kappa variable 3–20	IGKV3-20	−0.25	0.86	−3.08 to 2.57	0.28	0.84	−2.41 to 2.97	2.27	0.08	−0.24 to 4.79
Protein S100-A9	S100A9	3.4	0.03*	0.27 to 6.52	−1.48	0.34	−4.56 to 1.60	−0.63	0.66	−3.50 to 2.23
Talin-1	TLN1	1.02	0.47	−1.74 to 3.78	−1.95	0.14	−4.58 to 0.68	0.40	0.75	−2.04 to 2.83

The table shows the results of linear regression models between plasma proteins with clinical outcomes at follow-up. The models were adjusted for age, sex, and corresponding baseline protein levels. *PSS* Positive Symptom Severity score (based on the CAARMS assessment), *SOFAS* Social and Occupational Functional Assessment Scale, *BACS* composite score of Brief Assessment of Cognitive Function & *significant findings.

### Results from analysis 2: The association between omega-3 related plasma proteins and clinical outcome at 6-month follow-up

#### Association with positive symptom severity

In linear regression models, three plasma proteins at follow-up associated cross-sectionally with the PSS score at follow-up: Complement component 5 (C5), and protein S100A-9 showed a positive association (β coef = 3.54, CI 95%ile: 0.79 to 6.30, *p*-value = 0.01* & 3.40, CI 95%ile:0.27 to 6.52; *p*-value = 0.03*, respectively), while Immunoglobulin heavy constant gamma chain-4 (IGHG-4) showed an inverse association with the PSS score (β coef = −3.13, CI95%ile: −5.79 to −0.47 & *p*-value = 0.02*) (Table 3).

#### Association with functional outcome

Complement C5 associated inversely with the SOFAS score at follow-up (β coef = −3.23, CI95%ile = −5.87 to −0.59 & *p*-value = 0.02*). Apolipoprotein D (Apo D) at follow-up revealed a positive association with SOFAS score with a β coefficient of 2.77 (CI 95%ile: 0.13 to 5.41, *p*-value = 0.04*) (Table 3).

#### Association with cognition

In linear regression models, six proteins that are involved with the complement-coagulation cascade and lipid transport pathways associated with cognition. Among these, Complement Factor B (CFB) inversely associated with BACS score at follow-up (β coef = −3.18, CI 95%ile: 5.72 to −0.63 & *p*-value = 0.02), whereas Complement C1q subcomponent-B (C1QB) and coagulation factor V (F5) were positively associated with BACS score at follow-up (β coef = 3.93, CI95%ile: 1.58 to 6.28, *p*-value = 0.001* & β coef = 3.67, CI95%ile = 1.22 to 6.11; *p*-value = 0.004*). From the proteins involved in lipid transport mechanism, Apolipoprotein E, C-III and D were positively associated with BACS score (β coef = 3.09, 2.85, and 3.87; CI95%ile = (0.71 to 5.48), (0.36 to 5.34), and (1.25 to 6.50), *p*-value = 0.01*, 0.03*, and 0.004*, respectively) (Table 3).

### Results of analysis 3: Univariate mediation analysis

#### Positive symptom severity

In a univariate mediation model of C5, IGHG-4, and S100A49, total omega-3 did not exert any direct or total effect on PSS score at follow-up. However, C5 and S100A49 exerted a significant negative, indirect effect (mediation effect) on the relationship between change in total omega-3 PUFAs and PSS score at follow-up [β coef = −0.21 and −0.18; 95%ile CI = (−0.46 to −0.03) and (−0.42 to −0.01)] (Table 4 and Fig. 1).

**Table 4:** Results of univariate mediation analysis of omega-3 fatty acid associated plasma proteins on clinical outcome at follow-up.

Outcome	Mediator	Mediation effect β coef (95%ile CI)	Direct effect β coef (95%ile CI)	Total effect β coef (95%ile CI)
PSS	Complement C5	−0.21* (−0.46 to −0.03)	−0.06 (0.85 to −0.70)	−0.27 (0.39 to −0.90)
	Protein S100-A9	−0.18* (−0.42 to −0.01)	−0.09 (−0.78 to 0.73)	−0.27 (0.38 to −0.90)
	Immunoglobulin heavy constant gamma 4	−0.17 (−0.46 to 0.029)	−0.09 (−0.72 to 0.54)	−0.26 (−0.88 to 0.37)
SOFAS	Complement C5	0.19* (0.006 to 0.42)	0.11 (−0.51 to 0.73)	0.30 (−0.30 to 0.90)
	Apolipoprotein D	0.15 (−0.004 to 0.34)	0.20 (−0.42 to 0.82)	0.34 (−0.25 to 0.95)
BACS	Complement factor B	0.11 (−0.02 to 0.31)	0.54 (−0.04 to 1.13)	0.65* (−0.07 to 1.24)
	Complement C1q subcomponent subunit B	0.24* (0.05 to 0.54)	0.38 (−0.19 to 0.95)	0.62* (0.06 to 1.18)
	Coagulation factor V	0.18* (0.02 to 0.38)	0.47 (−0.10 to 1.05)	0.66* (0.09 to 1.23)
	Apolipoprotein E	0.16 (−0.01 to 0.43)	0.46 (−0.11 to 1.03)	0.62* (0.06 to 1.18)
	Apolipoprotein C-III	0.14 (−0.02 to 0.39)	0.52 (−0.06 to 1.10)	0.66 (−0.10 to 1.23)
	Apolipoprotein D	0.15 (−0.02 to 0.33)	0.51 (0.08 to −0.06)	0.67 (0.10 to 1.24)

The table shows the results of mediation analysis using change in omega-3 PUFAs, plasma proteins and clinical outcomes as exposure, mediator and outcome variables, respectively. The model is adjusted for age, sex and baseline total omega-3 levels. *CI* confidence interval, *PSS* Positive Symptom Severity score, *SOFAS* Social and Occupational Functional Assessment scale, *BACS* Brief Assessment of Cognitive Function & *significant findings.

The bold values in the table represent the β coefficient and confidence intervals of significant proteins.

#### Functional outcome

For SOFAS score at follow-up, no direct or total effect was observed for total omega-3 PUFAs. However, Complement C5 showed a significant positive mediation effect on the relationship of change in total omega-3 PUFAs on the SOFAS score at follow-up [β coef = 0.19; 95%CI = (0.01 to 0.42)] (Table 4).

#### Cognitive outcome

Univariate mediation analysis was developed for six plasma proteins (FB, C1QB, F5, Apo E, Apo CIII & Apo D). A significant positive total effect was observed for total omega-3 PUFAs associated with cognitive outcome. C1QB and F5 exerted a significant positive mediation effect on total omega-3 PUFAs related cognitive improvement [β coef = 0.24 & 0.18; 95%CI = (0.05 to 0.54) & (0.02 to 0.38)] (Table 4). This mediation effect of C1QB and F5 was found to be 39 and 27% of the total effect of total omega-3 PUFAs on cognition, respectively.

#### Role of baseline total omega-3 PUFAs on the mediation effect

In this model, no total effect was observed for change in total omega-3 PUFAs on any of the clinical outcomes. However, the mediation effect of complement and coagulation proteins on total omega-3 associated clinical outcome remained significant after adjusting the models for baseline total omega-3 PUFAs (Supplementary Table 4).

## Discussion

The current study investigated both the biological and clinical effect of PUFAs in a clinical high-risk population for a first psychotic episode. We have previously shown in this sample that supplementation with fish oil can have beneficial clinical effects in CHR individuals [63]. Our findings now provide evidence for a plausible mechanism of action of omega-3 PUFAs in early psychosis patients. The mass-spectrometry-based exploration of the plasma proteome at baseline and follow-up time points enabled us to study the longitudinal relationship of total omega-3 PUFAs on various biological mechanisms associated with psychopathology of CHR participants. First, change in total omega-3 PUFAs was associated with plasma proteins that represent inflammation, clotting and vesicle mediated transport mechanisms in CHR participants (Table 3). Secondly, among the omega-3 PUFA associated proteins, those participating in immune pathways of the complement system (C5, CFB, C1QB, and S100A9), the coagulation pathway (F5) and lipid transport pathways (Apo E, Apo CIII, and Apo D) were significantly associated with clinical outcomes. Thirdly, the results of the mediation analysis demonstrated that omega-3 PUFAs might exert a beneficial clinical response through immune pathway proteins (mainly the complement and coagulation cascade). There was evidence that C5 and S100A9 mediated the association of change in total omega-3 PUFAs with reduction in positive symptom severity and improvement in functioning. Furthermore, the proteins F5 and C1QB mediated the association between change in total omega-3 PUFAs and cognitive improvement at follow-up.

The current study is the first to observe that the complement cascade as the top biological pathway is related with change in omega-3 PUFAs in a CHR population. These observations provide vital evidence in omega-3-based treatment response in psychosis for the following reasons: (i) Genetic studies have reported evidence of a potentially causal relationship between increased long chain PUFA concentrations and lowered risk of psychosis [64, 65]; (ii) Complement related immune activity has been found to be involved in the pathophysiology of schizophrenia [66–70]; and (iii) in rodents, Madore et al. found a link between maternal omega-3 PUFA and microglia associated synaptic pruning through complement protein activity in off-springs [15]. However several studies have observed the beneficial effects of omega-3 PUFAs in various inflammatory and metabolic conditions [14, 15, 71–74], one recent study by Manousopoulou et al., explored the influence of dietary intake of omega-3 PUFAs on the plasma proteome of patients with fatty liver disease. The study reported that the coagulation pathway was highly influenced by the intake of marine omega-3 PUFAs in patients compared with healthy controls [75]. Whereas in the current study the inter-individual heterogeneity was taken into account by quantifying the proteins in each sample. Moreover, in the present study, erythrocyte membrane omega-3 level was used, which not only reflects the dietary intake of omega-3 PUFAs but also indicates the neuronal membrane omega-3 PUFAs [54, 55, 76, 77].

**Fig. 2:**
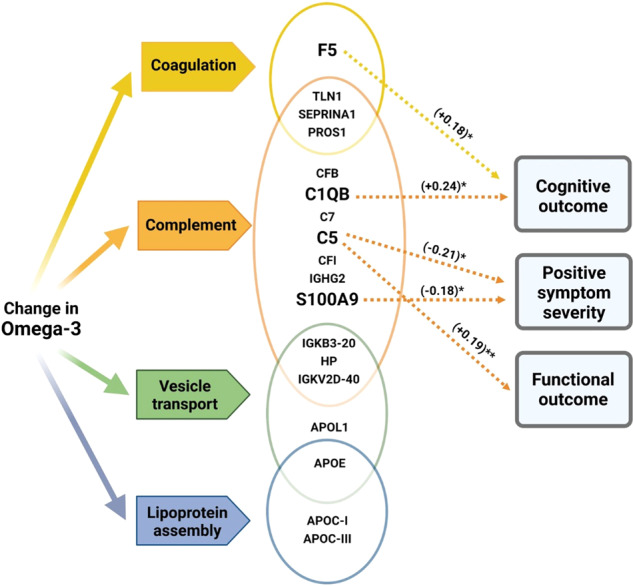
Schematic representation of key results depicting the relationship of total omega-3 PUFAs, key plasma proteins/pathways, and clinical outcome in a clinically high-risk population.

In line with our findings, existing evidence indicates consistent associations of complement dysregulation with psychotic symptoms in early psychosis although this has not be seen in established schizophrenia [78–84]. Complement and coagulation pathway proteins (that were associated with omega-3 PUFAs) indicated a relationship with psychotic symptoms (PSS), functioning status (SOFAS), and cognitive symptoms (BACS), whereas lipoprotein assembly proteins associated with cognition and functional outcome. The mediation analysis revealed a potential molecular mechanism through which total omega-3 PUFAs could influence clinical outcomes (as measured by PSS, a positive symptom score calculated based on CAARMS score), SOFAS (functioning), and BACS scales (cognition) (Fig. 2). Complement protein C5 mediated the association between change in total omega-3 PUFAs with reduction in positive symptoms and improvement in functional outcome in CHR. S100A9 which can regulate the expression of C3 [85, 86] mediated the omega-3 PUFA-associated reduction in PSS. In the mediation models, no direct association (no direct effect) was obtained for change in total omega-3 PUFAs (exposure) with clinical outcomes. However, a significant indirect association (indirect effect) was observed for change-in omega-3 PUFAs with clinical outcomes through the mediators (C5 and S100A9 for PSS; C5 for SOFAS score). Such type of mediation without direct effect is called as indirect-only mediation [87]. In mediation models of cognitive outcome, a significant direct and mediating effect was observed. Importantly, an increase in omega-3 PUFAs significantly associated with a high cognitive score at follow-up in which complement and coagulation proteins (C1QB and F5) exerted a partial mediation effect. Such mediation is termed ‘complementary mediation’, where the mediators (plasma proteins) complement the association of omega-3 PUFAs on cognitive outcome [87]. This partial mediation of C1QB and F5 contributed approximately 33 and 27% of the total effect of change in total omega-3 PUFAs on cognition. The latter mediation effect did not change even when adjusting the model for baseline omega-3 FAs. Previous investigations in the same cohort have reported significant cross-sectional and longitudinal associations of omega-3 PUFAs (EPA and DHA) on plasma immune markers, although these associations did not indicate any clinical significance [47]. Hence, our results from the mediation analysis suggest that omega-3 PUFAs associated changes in complement and coagulation proteins (F5, C1QB, C5, and S100A9) partially mediate the clinical response in a CHR state.

The key proteins that indicated a mediation effect were previously found to be involved with neuronal development and functioning [88, 89]. The activated products of C5, namely C5a, and S100A9, are pro-inflammatory in nature and play crucial roles in neuronal progenitor cell proliferation [90–92]. In our study, the mediation analysis suggests that an increase in total omega-3 PUFAs leads to symptomatic improvement by reducing the potentially pro-inflammatory components (C5 & S100A9). Similarly, the mediation analysis suggested that total omega-3 PUFAs improve cognition by increasing proteins (C1QB) that are involved in the synaptic pruning process [93, 94]. The animal study by Madore et al. provided a similar relationship of omega-3 PUFAs-C1Q-cognition axis. Madore et al.’s study reported that C1Q-receptor level was reduced in omega-3 deficient animals resulting in cognitive impairment [15]. Such symptom-specific complement alterations in a psychiatric population unfolds novel therapeutic opportunities to consider complement-targeted medicine in the early intervention of psychosis.

Our study has several strengths: (i) a state-of-the-art discovery proteomic approach allowed us to investigate a wide range of molecular mechanisms from plasma samples, (ii) the availability of biological and clinical data of the NEURAPRO clinical trial enabled us to look at both the biological and clinical relationship of omega-3 PUFAs at the same time, (iii) the exposure variable “erythrocyte membrane total omega-3 PUFA levels” provided a reliable measure of dietary omega-3 PUFAs and neuronal membrane omega-3 PUFAs [95], (iv) we used a unique study population (CHR) with mild psychotic symptoms, functional decline and cognitive impairment with no exposure to anti-psychotic medication [51], (v) the availability of both erythrocyte total omega-3 PUFAs and plasma proteome data at two time points provided the possibility of analyzing the longitudinal biological effects in the study population, and (vi) the findings have important clinical implications to early intervention strategies in psychosis.

Our study is not without limitations. Firstly, in the statistical analysis, the results were not adjusted for multiple corrections mainly due to the exploratory nature of the analysis and the nature of the mass spectrometry, which is a DDA based discovery approach. Secondly, in the statistical analysis, we adjusted the models for age and sex. The association of other covariates such as BMI and exposure to anti-depressants on both biological and clinical variables is not clearly understood and hence was not considered in the analysis. Finally, the absence of a direct effect in the mediation analysis limited us from understanding the percentage contribution of mediation in the overall effect [87, 96].

In conclusion, our findings provide novel insights into omega-3 PUFA-related protein mechanisms in the psychopathology of CHR participants. Pathway analysis indicated that the complement cascade showed the strongest association with change in omega-3 PUFAs. Furthermore, current findings suggest that the impact of omega-3 PUFAs on clinical symptoms in psychosis is mediated, at least in part, through complement and coagulation pathway proteins. For positive symptoms and functional outcome, the complement cascade proteins C5 and S100A9 exerted an ‘indirect-only mediation’ effect. Whereas for cognitive outcome, complement and coagulation pathway proteins (C1QB and F5) expressed a ‘complementary mediation’ effect. We speculate that omega-3 PUFAs may improve PSS and functional status through anti-inflammatory properties and enhance cognition by modifying C1Q-mediated synaptic pruning. Our study opens future opportunities to investigate the immune-associated intervention strategies in psychosis mainly targeting complement pathway proteins. Omega-3 PUFA are safe and can easily be used even in primary care settings. Since no biological treatment has yet been firmly established in CHR patients [97], the current findings also have potential implications for early intervention and treatment guidelines.

## Supplementary information

Supplementary materials and methods
