# Supplementary material for: Evidence that complement and coagulation proteins are mediating the clinical response to omega-3 fatty acids: A mass spectrometry-based investigation in subjects at clinical high-risk for psychosis
Source: Transl Psychiatry. 2022 Oct 28;12:454. doi: 10.1038/s41398-022-02217-0 (PMC9616837; doi:10.1038/s41398-022-02217-0)
Supplement: Supplementary file 1 — Supplementary materials and methods [file 41398_2022_2217_MOESM1_ESM.docx]

Supplementary Methods: Mass spectrometry based proteomic measures

Plasma samples of baseline and follow-up time points were processed according to the manufacturer’s instructions (PreOmics iST kit, no.iST 96x). Briefly, 4 µl of individual samples were solubilized in 50 μL of “Lyse” buﬀer (containing Tris-HCl, sodium deoxycholate (SDC), 0.1% sodium dodecyl sulfate (SDS), tris (2-carboxyethyl) phosphine (TCEP), and 2-chloroacetamide and heated to 95 °C for 10 min. 50 μL of the resulting denatured, reduced, and alkylated solution was transferred to the reaction tube. Enzyme (LysC and trypsin) was added, and samples were hydrolysed at 37°C for 1.5 hours. The resulting peptide mixture was washed and eluted as per the manufacturer’s instructions. The eluted peptides were vacuum-dried and dissolved in 100 µl of LC Load buffer. The reconstituted digested peptide mixture [200 ng/ µl] was then eluted using Evotips and injected using Evosep One (Evosep, Odense, Denmark ^96^. The digested samples were run on a Bruker timeTof Pro mass spectrometer connected to a Evosep One liquid chromatography system. The mass spectrometer was operated in positive ion mode with a capillary voltage of 1500 V, dry gas flow of 3 l/min and a dry temperature of 180^o^C. Trapped ions were selected for ms/ms using parallel accumulation serial fragmentation (PASEF). A scan range of (100-1700 m/z) was performed at a rate of 10 PASEF MS/MS frames to 1 MS scan with a cycle time of 1.15s^97, 98^. The MS raw files were then processed with MaxQuant^99^ version 1.6.17.0 as described in^98^ and the peptide data was further annotated and interpreted using the Perseus platform (V 1.6.7, [www.maxquant.net/perseus/](http://www.maxquant.net/perseus/))^100^. FDR was set at 0.01 to global protein identification level. Proteins that were identified in less than 70% of the total samples were not taken forward for analysis. Log_2_ transformed values of LFQ intensities were used for statistical analysis. Missing values of mass spectrometry based proteomic data (corresponding to values below the level of detection) were imputed with minimum values.

Supplementary Table 1: Results showing the list of plasma proteins associated significantly with change in omega-3 PUFAs (adjusted for age, sex and baseline total omega-3 levels)

| Protein Names | Coef. | P value | [95% Conf. Interval] | |
| --- | --- | --- | --- | --- |
| Alpha-1-antitrypsin | -1.05 | 0.01* | -1.82 | -0.27 |
| Alpha-1B-glycoprotein | -1.06 | 0.01* | -1.83 | -0.3 |
| Apolipoprotein C-I | -0.88 | 0.02* | -1.64 | -0.12 |
| Apolipoprotein C-III | 1.36 | <0.01* | 0.61 | 2.12 |
| Apolipoprotein D | 0.9 | 0.03* | 0.1 | 1.7 |
| Apolipoprotein E | 1.03 | 0.01* | 0.28 | 1.77 |
| Apolipoprotein L1 | -1.45 | <0.01* | -2.18 | -0.72 |
| Caspase-14 | -0.89 | 0.02* | -1.65 | -0.12 |
| Coagulation factor V | 0.9 | 0.02* | 0.14 | 1.66 |
| Complement C1q subcomponent subunit B | 1.18 | <0.01* | 0.44 | 1.91 |
| Complement C5 | -1.13 | <0.01* | -1.88 | -0.37 |
| Complement component C7 | 0.83 | 0.03* | 0.07 | 1.6 |
| Complement factor B | -0.87 | 0.02* | -1.62 | -0.12 |
| Complement factor I | -0.78 | 0.04* | -1.54 | -0.03 |
| Filamin A-interacting protein 1-like | -0.96 | 0.01* | -1.73 | -0.2 |
| Galectin-3-binding protein | -1.28 | <0.01* | -2.05 | -0.52 |
| Haptoglobin | -0.97 | 0.01* | -1.72 | -0.22 |
| Immunoglobulin heavy constant gamma 2 | 0.93 | 0.02* | 0.17 | 1.7 |
| Immunoglobulin heavy constant gamma 4 | 0.98 | 0.01* | 0.23 | 1.72 |
| Immunoglobulin heavy variable 1-18 | 0.82 | 0.03* | 0.07 | 1.58 |
| Immunoglobulin heavy variable 3-7 | -0.8 | 0.04* | -1.55 | -0.05 |
| Immunoglobulin kappa variable 3-20 | 1.05 | 0.01* | 0.3 | 1.81 |
| Protein S100-A9 | -1.03 | 0.01* | -1.78 | -0.29 |
| Talin-1 | -0.92 | 0.02* | -1.68 | -0.17 |

The table shows the results of linear regression models between change in total omega-3 PUFAs and plasma proteins at follow-up. The models were adjusted for age, sex and baseline total omega-3 levels.

Supplementary Table 2: Results of linear regression model showing the list of plasma proteins associated significantly with change in omeha-6 PUFAs (adjusted for age, sex and baseline omega-6 levels)

| Protein names | Coef. | P value | [95% Conf.Interval] | |
| --- | --- | --- | --- | --- |
| Apolipoprotein C-I | 0.8 | 0.02 | 0.12 | 1.48 |
| Apolipoprotein L1 | **1.05** | **0.00** | **0.39** | **1.72** |
| Hemoglobin subunit beta | 0.79 | 0.03 | 0.08 | 1.49 |
| Immunoglobulin lambda variable 1-36 | -0.82 | 0.02 | -1.5 | -0.14 |
| Protein S100-A9 | 0.78 | 0.02 | 0.1 | 1.46 |
| Vitamin D-binding protein | -0.84 | 0.02 | -1.53 | -0.15 |

The table shows the results of linear regression models between change in omega-6 PUFAs and plasma proteins at follow-up. The models were adjusted for age, sex and baseline omega-6 levels.

| Pathway name | #Entities found | #Interactors found | #Reactions found | Entities pValue | Entities FDR |
| --- | --- | --- | --- | --- | --- |
| Scavenging of heme from plasma | 2 | 0 | 5 | 0.00 | 0.06 |
| Binding and Uptake of Ligands by Scavenger Receptors | 2 | 0 | 5 | 0.00 | 0.06 |
| Inhibition of nitric oxide production | 0 | 1 | 1 | 0.00 | 0.06 |
| VLDL clearance | 1 | 0 | 3 | 0.00 | 0.06 |
| Metal sequestration by antimicrobial proteins | 1 | 0 | 2 | 0.00 | 0.06 |
| Erythrocytes take up oxygen and release carbon dioxide | 1 | 0 | 1 | 0.00 | 0.06 |
| Erythrocytes take up carbon dioxide and release oxygen | 1 | 0 | 2 | 0.01 | 0.06 |
| O2/CO2 exchange in erythrocytes | 1 | 0 | 3 | 0.01 | 0.06 |
| IRAK4 deficiency (TLR2/4) | 1 | 0 | 2 | 0.01 | 0.09 |
| Neutrophil degranulation | 2 | 0 | 3 | 0.01 | 0.09 |
| MyD88 deficiency (TLR2/4) | 1 | 0 | 2 | 0.01 | 0.10 |
| Transport of small molecules | 2 | 1 | 11 | 0.01 | 0.10 |
| Regulation of TLR by endogenous ligand | 1 | 0 | 1 | 0.01 | 0.10 |
| VLDL assembly | 1 | 0 | 1 | 0.02 | 0.10 |
| Vesicle-mediated transport | 2 | 1 | 9 | 0.02 | 0.12 |
| Plasma lipoprotein clearance | 1 | 0 | 3 | 0.03 | 0.12 |
| Thrombin signalling through proteinase activated receptors (PARs) | 0 | 1 | 2 | 0.03 | 0.12 |
| Autophagy | 1 | 1 | 13 | 0.03 | 0.12 |
| Late endosomal microautophagy | 1 | 0 | 3 | 0.03 | 0.12 |
| Plasma lipoprotein assembly | 1 | 0 | 1 | 0.03 | 0.12 |
| Post-translational protein phosphorylation | 1 | 0 | 1 | 0.03 | 0.12 |
| Mitochondrial Fatty Acid Beta-Oxidation | 0 | 1 | 1 | 0.04 | 0.12 |
| Defective CFTR causes cystic fibrosis | 0 | 1 | 2 | 0.04 | 0.12 |
| Antimicrobial peptides | 1 | 0 | 2 | 0.04 | 0.12 |
| ABC transporter disorders | 0 | 1 | 2 | 0.04 | 0.12 |
| WNT5A-dependent internalization of FZD4 | 0 | 1 | 2 | 0.05 | 0.12 |
| PINK1-PRKN Mediated Mitophagy | 0 | 1 | 2 | 0.05 | 0.12 |

Supplementary Table 3: Pathways significantly associated with 6-month change in omega-6 PUFAs

Supplementary Table 4: Results of mediation analysis adjusted for baseline total omega-3 PUFAs in addition to age, sex and baseline protein levels

| Outcome | Mediator | Mediation effect | Direct effect | Total effect |
| --- | --- | --- | --- | --- |
| SOFAS | C5 | 0.23 *  (0.03 to 0.50) | -0.06  (-0.68 to 0.56) | 0.17  (-0.44 to 0.78) |
|  | APOD | 0.09  (-0.04 to 0.25) | 0.12  (-0.50 to 0.74) | 0.21  (-0.40 to 0.82) |
| BACS | CFB | 0.10  (-0.02 to 0.31) | 0.47  (-0.14 to 1.07) | 0.57  (-0.04 to 1.17) |
|  | C1QB | 0.29 *  (0.06 to 0.63) | 0.24  (-0.35 to 0.83) | 0.53  (-0.05 to 1.11) |
|  | Factor V | 0.16 *  (0.02 to 0.35) | 0.42  (-0.17 to 1.00) | 0.58  (-0.04 to 1.17) |
|  | APOE | 0.20  (-0.00 to 0.48) | 0.37  (-0.22 to 0.97) | 0.57  (-0.01 to 1.15) |
|  | APOC3 | 0.18  (-0.02 to 0.43) | 0.40  (-0.21 to 1.00) | 0.58  (-0.001 to 1.16) |
|  | APOD | 0.10  (-0.01 to 0.26) | 0.48  (-0.10 to 1.07) | 0.59  (-0.001 to 1.17) |
| PSS | C5 | 0.23 *  (-0.49 to -0.36) | -0.08  (-0.74 to 0.58) | -0.31  (-0.96 to 0.34) |
|  | S100A9 | 0.18 *  (-0.44 to -0.01) | -0.11  (-0.77 to 0.55) | -0.29  (-0.93 to 0.35) |
|  | IGHG4 | -0.19  (-0.49 to 0.04) | -0.12  (-0.72 to 0.77) | -0.30  (-0.94 to0.34) |

The table shows the results of mediation analysis using change in total omega-3 PUFAs, plasma proteins and clinical outcomes as exposure, mediator and outcome variables, respectively. The model is adjusted for age, sex, baseline total omega-3 levels and baseline total omega-3 PUFA levels.CI- confidence interval, PSS- Positive Symptom Severity score, SOFAS- Social and Occupational Functional Assessment scale, BACS- Brief Assessment of Cognitive Function & *significant findings
